# Application of infrared thermography for estimating residual stress in ground anchors for maintenance

**DOI:** 10.1038/s41598-022-27222-7

**Published:** 2023-01-02

**Authors:** Dae-Hong Min, Byeong-su Jang, Hyung-Koo Yoon

**Affiliations:** Department of Construction and Disaster Prevention Engineering, Daejeon University, Daejeon, 34520 Korea

**Keywords:** Civil engineering, Structural materials

## Abstract

Characterizing the integrity of ground anchors is essential for examining their usability in the maintenance of soil structure. However, the lift-off test, which is generally used for this purpose, has limitations when applied to covering all installed ground anchors. The objective of this study is to assess the possibility of using infrared thermography to measure the residual stress in ground anchors as a noncontact technique that bypasses the disadvantages associated with existing techniques. A preliminary experiment is performed to determine the exact emissivity of the tested materials. Both passive and active methods, as representative techniques in infrared thermography, are applied. In the large-scale experiment, infrared images of four installed strands with growing stress in the range of 0–400 kPa at 100 kPa intervals are used in the passive method of measurement. For the active method, these same stress ranges are applied to a heated anchor head using a UTM machine. The results of the passive method show that the temperature increased and decreased according to load and unload steps. Values for the cooling rate index are deduced through the active method results, and reliable behavior are observed at 10 and 15 min. The number of pixels with huge temperature changes also changed with the loading step in both passive and active methods. This study demonstrates that infrared thermography is a suitable alternative method for assessing the residual stress in ground anchors as a type of noncontact technique.

## Introduction

Ground anchors provide a high load capacity for stabilizing soil structure in constructions on slopes, and regular inspections are required to estimate the performance in deteriorating and aging anchors^1,2^. The lift-off test is generally used to estimate the residual stress of anchors^3,4^, wherein the anchor head is pulled out using a hydraulic jack, however, this method has limitations in application as it is expensive and time consuming^5^. It is impossible to investigate all anchors constructed in the same site, and thus, residual stress is usually only evaluated in a few anchors through sampling tests. A nondestructive technique has been proposed to evaluate the performance of a sufficient number of anchors. Zima and Rucka^6^ attempted to inspect stress characterization in differently fixed lengths of ground anchors based on energy transfer generated by guided wave, wherein energy transfer is caused by a geometric parameter between the tendon and the surrounding anchor body. Tamrakar et al.^7^ used ultrasonic waves to deduce the residual stress in ground anchors, and both the waveform and maximum amplitude on the surface of bearing plates were determined. The generalized relationship between tensile stress and measured value was suggested through laboratory and field experiments. Frequency domain reflectometry was also selected for the characterization of an anchor rod buried in concrete beneath the ground, and this method also provides information about the deteriorated location for maintenance^8^. The proposed method is also cumbersome concerning achieving contact of the sensor on the anchor head. Thus, it is necessary to develop noncontact forms of maintenance methods. This study assesses the application of infrared thermography as a maintenance technique for estimating the stress level in ground anchors.

Infrared thermography (IRT) was initially used for military purposes^9,10^ and has been recently used in the engineering field to determine the characteristics and defects of objects^11–18^. Heat generated from an object depends on radiation, convection, and conduction, and among these, radiation has the greatest effect on the diffusion of thermal energy^19–21^. Thermal energy is emitted from all objects with a temperature condition over − 273 °C (0 K) at wavelengths in the range of 0.7–1000 μm. IRT is used to measure the thermal energy emitted from an object in given conditions, and the thermographic technique is divided into active, hybrid and passive methods for experiments and analysis^22^. The active method measures the temperature of an object in the heating or cooling state through an artificial heat source, and the passive method determines the temperature distribution of an object without using an additional heat source. Therefore, the active method is a quantitative analysis method, and the characteristics of the object can be understood through the relative comparison of measured values. In the passive method, qualitative analysis is performed after recognizing the abnormal state corresponding to the temperature in advance^23–25^. This paper attempts to derive a useful method for evaluating the performance of ground anchors and considers both active and passive methods.

This manuscript begins by explaining the engineering concept that allows predicting residual stress using the IRT technique, and the relationship between thermal expansion and stress is also expressed through a mathematical model. To suggest a reasonable method for evaluating residual stress of the anchor with IRT, the experimental procedures and results of active and passive methods are described. Based on this, the behavior of residual stress and temperature measured by infrared thermography is explained.

## Background theory

As the temperature of an object increases, the object emits more thermal flux. The thermal flux is defined as total energy radiated per unit surface in time, and it is referred to by various names, including black-body irradiance, energy flux density, radiant flux, or emissive power^26–28^. According to Stefan–Boltzmann law, thermal flux (I) is a function of temperature (T) and a proportional constant (μ). However, the formula for thermal flux is defined as Eq. (1), which considers the absorbed amount and emitted energy because an object does not absorb all of the radiated heat.

1 $$ {\text{I}} =\uppsi \cdot\upmu \cdot T^{4} $$

where the proportionality constant of μ is Stefan–Boltzmann coefficient 5.6704 × 10^–8^ W/m^2^ K, and ψ denotes the emissivity, which is the ratio of radiated energy with a black body to an object. The emissivity is theoretically a unit for a black body, and the value is smaller than 1 in natural condition^29^ because it is greatly affected by the molecular structure and surface characteristic of the target object. Even though reasonable emissivity is suggested according to materials, a preliminary test is necessary for estimating emissivity that meets the experimental conditions considering the wavelength of infrared light and the characteristics of air in the atmosphere^30^.

Thermoelasticity represents thermal energy generation according to the stress (load) change of an object^31,32^. In thermodynamic theory, stress (σ) is defined according to strain (ε) and temperature (T) as Eq. (2), and it is used to estimate the stress caused by thermal change.

2 $$ {\text{du}} =\upsigma _{{{\text{ij}}}} {\text{d}}\upvarepsilon _{{{\text{ij}}}} + {\text{Tds}}\quad {\text{i}},\;{\text{j}} = 1,2,3 $$

where u is the internal energy per unit volume, σ_ij_ and ε_ij_ are components of the stress and strain tensors, T is the absolute temperature, s is the entropy. With elastic solids in adiabatic condition, Eq. (3) can be obtained:

3 $$ {\rho C}_{\upvarepsilon } = - \left[ {\frac{{\partial {\text{C}}_{{{\text{ijkl}}}} }}{{\partial {\text{T}}}}\left( {\upvarepsilon _{{{\text{kl}}}} - \upalpha _{{{\text{kl}}}} {\Delta T}} \right) - {\text{C}}_{{{\text{ijkl}}}} \left( {\upalpha _{{{\text{kl}}}} + \Delta {\text{T}}\frac{{\partial \upalpha _{{{\text{kl}}}} }}{{\partial {\text{T}}}}} \right)} \right]{\text{d}}\upvarepsilon _{{{\text{ij}}}} $$

where ρ is the density, C_ε_ is the specific heat for constant strain, C_ijkl_ is the elastic tensor, αkl is the thermal expansion tensor. Then assuming C_ijkl_ and αkl are independent of T, the former equation can be simplified as:

4 $$ \Delta {\text{T}} = \frac{{{\text{T}}\upalpha _{{{\text{kl}}}} }}{{{\rho C}_{\upvarepsilon } }}{\text{C}}_{{{\text{ijkl}}}} {\text{d}}\upvarepsilon _{{{\text{ij}}}} . $$

For material with isotropic thermal expansion and elastic modulus. We can obtain the relationship between temperature difference and strain:

5 $$ \Delta {\text{T}} = \frac{{{\text{T}}\upalpha }}{{{\rho C}_{\upvarepsilon } }}\Delta\upvarepsilon _{{{\text{kk}}}} $$

where Δε_kk_ represents the sum of principal strain.

## Methodology

**Figure 1:**
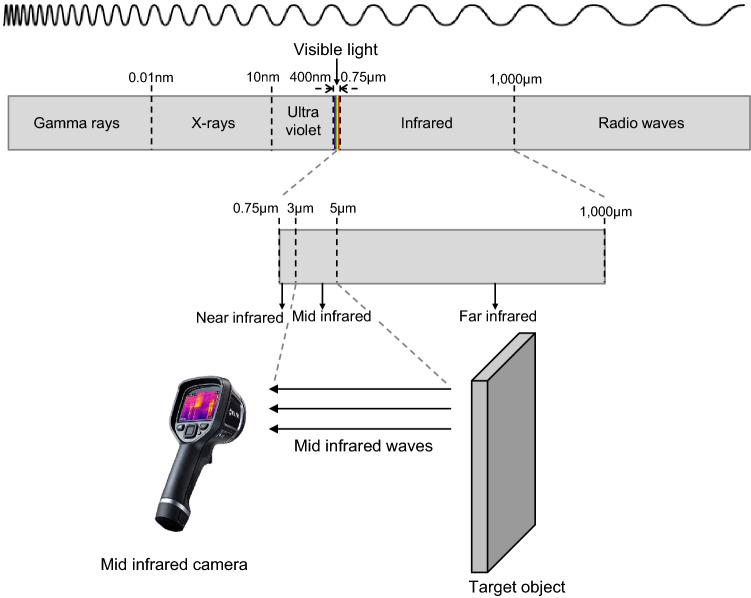
Principle of infrared thermography.

IRT obtains the thermal images through a thermal camera lens receiving light in the range of infrared wavelength (0.7–1000 μm), as shown in Fig. 1, and the type of thermal camera varies depending on the infrared wavelength range^33,34^. In this study, a mid-wavelength infrared (MWIR) camera was used, which measures thermal waves in the mid-wavelength range (3–5 μm). A thermal camera measures the temperature on the surface of an object, and the value can be distorted depending on the surface emissivity. In this study, the experiment for emissivity measurement of SM45C material, which is the same as used in ground anchors, was performed in advance to obtain a reliable temperature value. The thermal imaging camera made by FLIR was used, and it has 240 × 180 pixel resolution with a thermal sensitivity of 0.06 °C. In addition, it has a 15 fps sampling rate, meaning it can measure 15 frames per second.

### Emissivity

**Figure 2:**
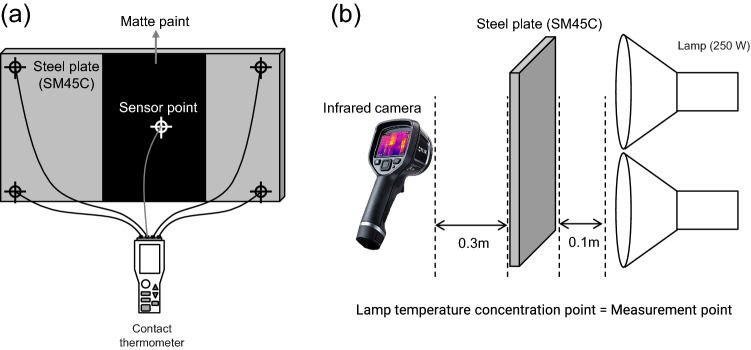
Experimental concept for deducing anchor head emissivity: (**a**) sensor location; (**b**) measurement.

The emissivity can be estimated through the radiated energy of an object as shown in Eq. (1); however, it has limitations for easily obtaining energy. The emissivity is used as an input value when measuring the temperature image of object, and thus, the value is estimated through difference between the measured thermal image and the actual temperature according to change of the input value. The emissivity was changed from 0.1 to 1.0, and the thermal image was measured. The anchor head can easily reflect the surrounding heat source since it is made of a steel material, and it is difficult to accurately measure the temperature change with a thermal camera. Therefore, matte paint (luminous intensity < 2%) was applied to the anchor head to minimize heat reflection, and the thermal properties were then observed. The thermal image was measured while raising the temperature using an infrared lamp on a steel plate made of the same material as the anchor head (SM45C) as shown in Fig. 2. The capacity of the infrared lamp was 250 W, and the distance from the steel plate to thermal imaging camera was maintained at about 0.3 m. The thermal energy was applied with an infrared lamp for 60 min until the temperature of the steel plate converged to a constant value of 27.5 °C in consideration of the laboratory temperature being about 20 °C. The contact thermometer was also attached to measure the exact temperature of the steel plate, and the emissivity showing a thermal image that approximates the actual temperature was inferred. The viewing angle of infrared camera set to measure the entire steel plate. In addition, the temperature was also measured with growing thickness of matte paint at 1 mm intervals in the range of 1–10 mm for determining the effect of thickness of the applied paint on emissivity.

### Passive method

**Figure 3:**
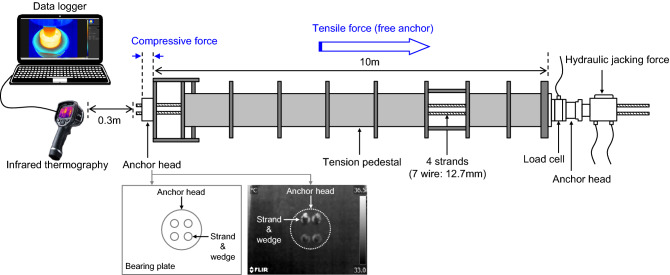
Ground anchor real scale infrared thermography experiment schematic.

The large-scale experiment was conducted to examine the relationship between residual stress and temperature based on the passive method, and it can simulate a tension seat with maximum load of 800 kN as shown in Fig. 3. The tensile force of 100 kPa can be applied to one strand (diameter: 12.7 mm, 7 wires), and thus, a total of 4 strands were installed; as a reference, the design tension of a ground anchor is generally 400 kPa. The length of the strand was set to 10 m in consideration of the length of the tension seat. The thermal measurement position was set to the fixing area where the anchor head is installed, and thus, the load-bearing device was installed in the opposite direction to the fixing area to prevent disturbance on measuring temperature. The tensile force was applied with a hydraulic jack (maximum capacity: 1 MN) and the load was checked through a load cell (maximum measuring range: 1 MN). The area of free anchor generates a tensile force when applying a load, though the compressive stress is applied to the anchor head. The load and unload pressure varied in the range of 0–400 kPa at 100 kPa intervals, and the thermal change was measured. The duration of each load step was 5 min, and the experiment was conducted for a total of 45 min. The thermal imaging camera was positioned 0.3 m away from the anchor head, and data were acquired at 15 frames per second (15 fps). The anchor head was set at room temperature of 28.5–29 °C for 24 h to reliably measure the temperature when the applied load is converted into thermal energy. The anchor head used the same model applied in the actual field, and the thickness, diameter, area, and elastic modulus were 43 mm, 108 mm, 0.0095 m^2^, and 2.06 × 1011 N/m^2^, respectively. The material of anchor head is SM45C, which comprises carbon, manganese, and silicon at 42–48%, 60–90%, and 0.15–0.35%, respectively. According to KS D-3752^35^, the yield load, tensile strength and elongation of SM45C are 490 N/mm^2^, 686 N/mm^2^ and 17% or more, respectively.

### Active method

**Figure 4:**
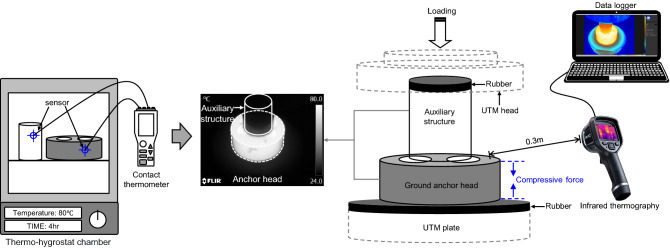
Schematic description for performing the active method using a heated anchor head and universal test machine (UTM).

In the active method, as shown in Fig. 4, artificial heat was applied to the anchor head to measure the temperature change according to the load. The anchor head was seated into a thermo-hygrostat with a temperature of 80 °C for 24 h to generate artificial heat. The experiment was designed to give the compressive force into anchor head, and thus, the universal test machine (UTM) (maximum capacity: 1 MN; minimum adjustable capacity: 0.1 kN) was selected. The load was adjusted in 5 steps of 0, 100, 200, 300, and 400 kPa in the same way as for the passive method. Measurements were carried out by monitoring the temperature decrease for about 1 h until the temperature of the heated anchor head converged to the laboratory temperature (25–28 °C). After that, the load was removed to examine whether there was any additional temperature change during 1 h. Rubber with low thermal conductivity was installed on the bottom of the anchor head to prevent the transfer of temperature generated by the UTM hydraulic to the anchor head. The thermal imaging camera was focused on the front of anchor head at a distance of 0.3 m, and data were acquired at 15 frames per second. The active method measures the cooling tendency of the artificially heated anchor head according to the load, and the unloading step was omitted because it limited the simulation of unloading conditions. The anchor head used the same model selected in the passive method.

## Results

### Emissivity

**Figure 5:**
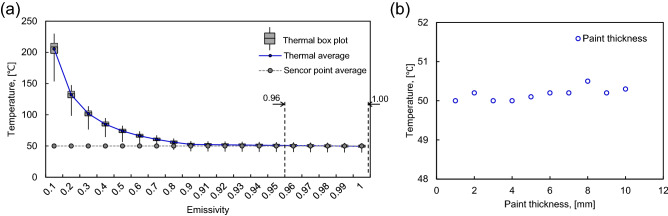
Deduced emissivity distributions for (**a**) comparison of values measured using thermometer versus infrared thermography; (**b**) various thicknesses of matte paint.

Figure 5a shows the box plot of temperature variation measured by the thermal imaging camera while changing the emissivity, and the temperature nonlinearly decreases as the emissivity increases. The ranges of box plot relatively huge in small emissivity of 0.1 and they were converged on the similar value of around 0.9 in emissivity. The emissivity was increased in 0.1 increments in the range of 0.1–0.9, and it was raised at 0.01 intervals in the range of 0.9–1.0 considering that the emissivity of typical paint is around 0.9^14,15^. When the emissivity was 0.1, the measured averaged temperature was recorded up to about 205 °C, which is significantly different from the actual temperature of 50 °C measured using a contact thermometer. The difference between the temperature measured by the thermal imaging camera and the contact thermometer decreases as the emissivity increases. The averaged gap shows 0.45 °C in the range of 0.96–1.00 of emissivity, which indicates excellent reliability. The emissivity of the matte paint was inferred to 0.96–1.00, and the average value of 0.98 was used as the emissivity of anchor head in this study. In addition, the temperature variation according to the paint thickness is shown in Fig. 5b, and the previously determined emissivity of 0.98 was used as an input value. Note that the temperature measured by the thermal imaging camera shows a constant value of 50 °C on average, regardless of thicknesses. The measured temperature is almost similar to the true value, and the thickness of the matte paint has little effect on the thermal image.

### Passive method

**Figure 6:**
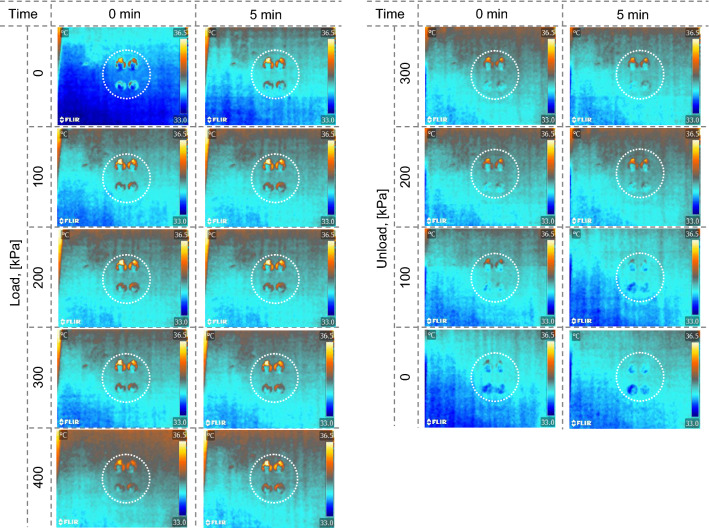
Thermal image measured by infrared thermography in the passive method.

**Figure 7:**
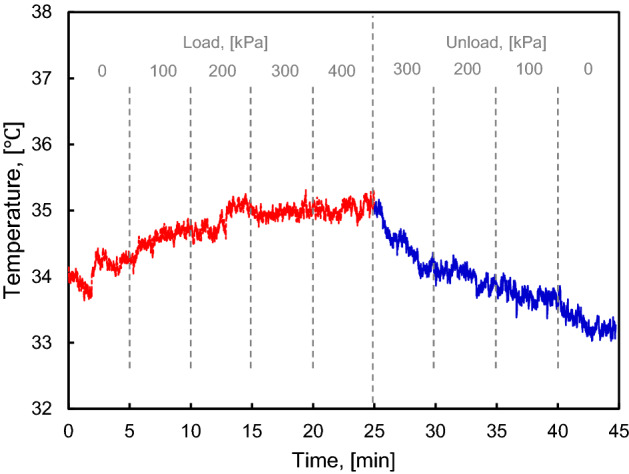
Average temperature change versus elapsed time at each loading step in the passive method.

The thermal image measured by the passive method is shown in Fig. 6, and the image is composed of 12,600 grids considering the resolution (240 × 180 pixels) of the thermal imaging camera used to observe the detailed characteristics. The images are arranged at 0 and 5 min for initial and final times of stress corresponding to the time interval of 5 min. The measured temperature range was 33.0–36.5 °C, and the anchor head was marked in white to distinguish it from the bearing plate. A relatively high temperature was measured at the location of the strand with application of load, and the temperature decreased at the same location during the unloading step. The temperature changes in the anchor head according to the loads were quantitatively averaged, and the results are shown in Fig. 7. The average value deviated slightly even under the same load, though the temperature increased ≈ 1 °C at 300 kPa compared with the initial temperature of 34 °C. At the last step of 400 kPa, the temperature difference from that at 300 kPa is an average of 0.03 °C, which shows that the temperature increase is minor. In the 300–400 kPa step, it is judged that the temperature change is low because the tension force of the hydraulic jack is not sufficiently transferred to the anchor head. In the unloading stage, the measured temperature generally decreased, and the final temperature was 33.3 °C, which is similar to the initial temperature. The reason that the temperature increases and decreases during loading and unloading steps is that the mechanical stress is converted into thermal stress based on thermodynamic theory, as shown in Eq. (2).

### Active method

**Figure 8:**
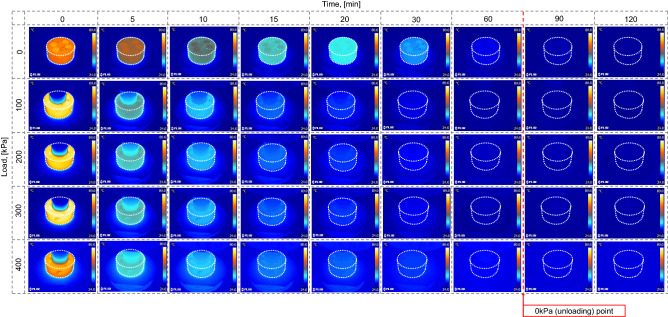
Thermal image measured by infrared thermography in the active method.

**Figure 9:**
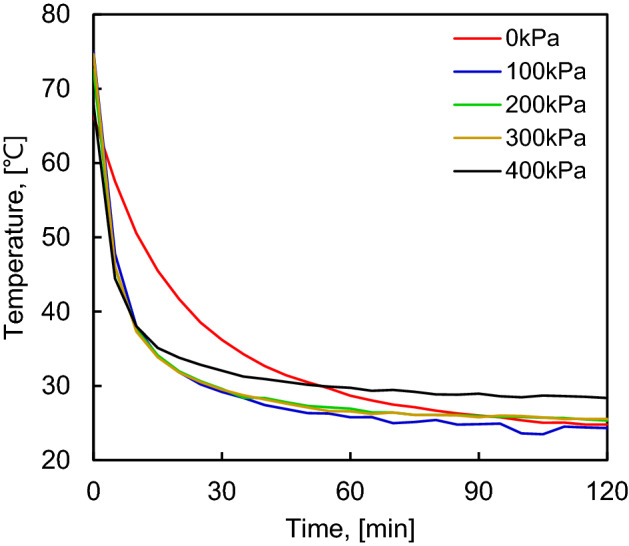
Average temperature change versus elapsed time at each loading step in the active method.

The data measured by the active method were imaged in the same way as for the passive method, and the results were collected for elapsed times of 0, 5, 10, 15, 20, 30, 60, 90, and 120 min. The values measured at 0 min were in the range of 69–80 °C because there is heating with a thermo-hygrostat chamber, and the temperature is rapidly reduced to 29–35 °C, which is approximately 45 °C of the change value at 30 min. In addition, the temperature converges to about 24 °C, which is similar to laboratory temperature, after 60 min with removal of the load to 0 kPa. It is noted that the temperature change effect on unloading was negligible. The temperatures at 9600 grids in Fig. 8 were quantitatively averaged and plotted as shown in Fig. 9. The temperature measured in the anchor head shows a nonlinear trend, with variation in the slope depending on the applied load. The heated anchor head at 0 kPa showed a relatively slower temperature change than other loads during 30 min, and the temperature continuously decreased even after 60 min. On the other hand, the anchor head loaded with 100, 200, 300, and 400 kPa shows that the temperature rapidly decreased up to about 10 min, and the temperature then converged to a constant value. Figure 9 shows the tendency of the temperature change during elapsed time, though it is limited with respect to determining the different behavior according to each load. The variation in slopes means that the cooling rates differ, and detailed analysis is necessary. Therefore, the content is specifically explained in the discussion section.

## Discussion

### Passive method

**Figure 10:**
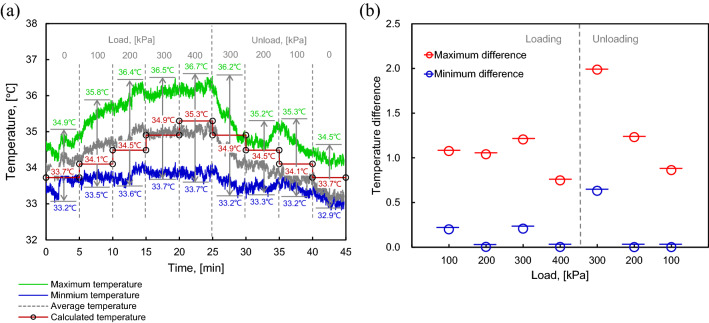
Distribution of measured temperature in the passive method: (**a**) maximum, minimum, and averaged temperatures; (**b**) temperature difference according to each load.

The extracted maximum, minimum, and average temperature from Fig. 6 are demonstrated in Fig. 10 according to elapsed time. Note that the temperature for 5 min fluctuates even with the same load. The maximum and minimum values ​​were selected according to the load to examine the entire range of the measured temperature. The maximum temperatures were estimated to 34.9, 35.8, 36.4, 36.5, 36.7, 36.2, 35.2, 36.3, and 34.5 °C for 0, 100, 200, 300, 400 (loading), 300, 200, 100, and 0 kPa (unloading). The lowest temperatures among the minimum values were selected as 33.2, 33.5, 33.6, 33.7, 33.7, 33.2, 33.3, 33.2, and 32.9 °C according to the loading and unloading order, respectively. The temperature changes of the maximum and the minimum values were calculated to 0.45 and 0.12 °C on average, respectively, in the loading stage. The unloading step also shows similar values of 0.55 and 0.20 °C on average for the maximum and minimum values, respectively. Although the temperature change was relatively small at around 300–400 kPa, it can be seen that reasonable temperature changes occur according to the loading and unloading steps.

The temperature theoretically calculated using Eq. (5) is shown in red color in Fig. 10a, which is used to verify the reliability of the measured temperature. The initial volume and thermal capacity of 393.72 cm^3^ and 0.486 J/K^36^, respectively, were used. The temperature reflects the temperature calculated for each load. The strain was calculated using the elastic modulus (2.06 × 10^11^ N/m^2^) of the anchor head, and thermal stress coefficient of 0.06 K was deduced considering the measured temperature. The theoretically calculated temperature is located near the average value, and the tendency increases and decreases as a function of loading and unloading steps. Note that the temperature measured by the thermal image is reliable, and the residual stress of the anchor head can be estimated using the passive method.

**Figure 11:**
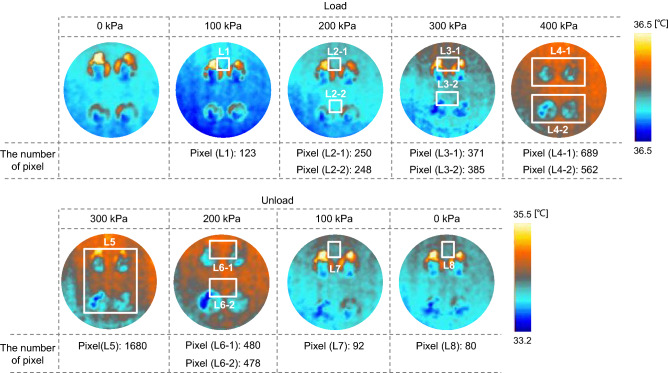
Thermal images measured by the passive method. The white box denotes the area of significant variation.

The maximum and minimum temperature difference for each load is shown in Fig. 10b to observe the change in the measured temperature according to the residual stress. The maximum temperature difference is calculated to 1.9 °C when unloading from 400 to 300 kPa. The minimum temperature difference is approximately 0.05 °C with both loading conditions of 200 and 400 kPa and unloading conditions of 200 and 100 kPa. Note that the load is differently transmitted for each location, and this study focused on the highly propagated areas of load. The position of the largest temperature change is indicated by a white box in Fig. 11, and the box appeared around the strand to which the compressive force was directly applied. Although the temperature change was observed the only in the upper strand when the applied load was 100 kPa, the enlargement of the white box to upper and lower strands shows growing load. Therefore, the number of pixels was 123 at 100 kPa, and the pixel area increased by 1251 (top: 689, bottom: 562) at 400 kPa. A similar behavior is observed for unloading conditions, though a relatively large temperature change was observed in 80 pixels due to residual stress even when all loads were unloaded at 0 kPa. Note that the initial load acts intensively on a part of the strand as a concentrated load, and the load changed to a distributed load on the front of the anchor head with growing load. In addition, it is considered that the load is dissipated during unloading, and the affected area is also reduced. The location, where the strand is installed, is also provided in the results, which show better prediction of the load change using the passive method.

### Active method

To quantitatively analyze the slope of the time–temperature graph in the active method results, the cooling rate index (CRI) was derived as the ratio of temperature change (ΔT_anchor_) per unit time (Δt) in Eq. (6)^37,38^.

6 $$ CRI_{min} = {{\Delta T_{anchor} } \mathord{\left/ {\vphantom {{\Delta T_{anchor} } {\Delta t}}} \right. \kern-0pt} {\Delta t}} $$

**Figure 12:**
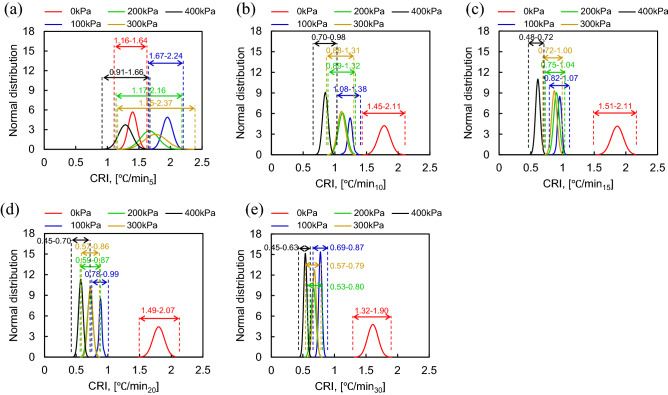
Converted cooling rate index (CRI) based on the active method with time intervals of (**a**) 5 min; (**b**) 10 min; (**c**) 15 min; (**d**) 20 min; (**d**) 30 min.

**Figure 13:**
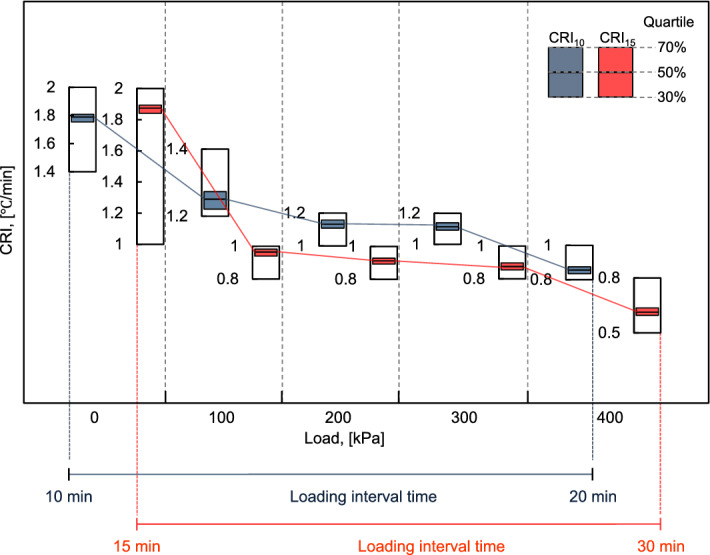
Box plots of CRI10 and CRI15 based on quartiles.

**Figure 14:**
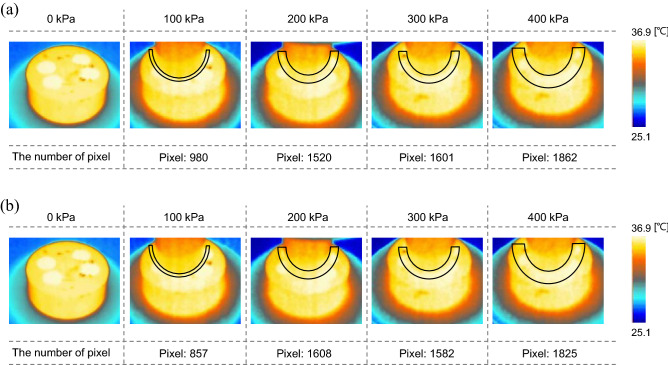
Thermal images measured by the active method through: (**a**) CRI10; (**b**) CRI15. The black box denotes the area of significant variation.

The unit time was set to 5, 10, 15, 20, and 30 min to examine CRI distributions for distinguishing values from each load. The curve of calculated CRI is demonstrated to be normally distributed, as shown in Fig. 12, and the ranges of the maximum and minimum CRI are also indicated. The CRI_5_ calculated for 5 min shows a large overlapping area of each curve, and it is difficult to separate the load with CRI_5_. However, a smaller temperature change occurred with applied load in CRI_10_, CRI_15_, CRI_20_, and CRI_30_. The distributed load is generally applied into the anchor head when increasing load, as shown in the results of the passive method with expanded pixel area. This is related to the frequency of the normal distribution curve, and thus, the peak value of the normal distribution curve should be higher as the load increases. CRI_10_ and CRI_15_ satisfy the conditions, and the results of CRI_10_ and CRI_15_ are thus shown to be the best for the active method. Additionally, the overlapping area of each CRI should be minimized to predict the appropriate load. The CRI_10_ and CRI_15_ were distributed in the range of 20–80% quantiles, and the values are summarized in Table 1. The quantile values of 30–70% show relatively small overlapping areas in CRI_10_ and CRI_15_, which are indicated in green and red, respectively. Although CRI_10_ showed a slight overlap in data between 200 and 300 kPa for residual stress, there is no overlapping regions for the remaining loads in CRI_10_ and CRI_15_. Therefore, the CRI_10_ and CRI_15_ can provide reasonable estimates of residual stress with the 30–70% confidence interval using the active method. The CRI_10_ and CRI_15_ corresponding to the confidence interval are plotted in Fig. 13, and the average values are shown as a line. Although the ratio of change in the CRI for each load is different, it is possible to classify the load by adjusting the confidence interval with CRI_10_ and CRI_15_ as described above. The pixel location showing the maximum temperature difference based on the CRI_10_ and CRI_15_ is demonstrated in Fig. 14, analyzed with a confidence interval of 30–70%. The position where the ratio of temperature change is high is focused on the center of the anchor head, where the UTM machine was applied the load. Both CRI_10_ and CRI_15_ show that the number of temperature difference pixels increased to 190% and 210%, respectively, along with a growing load from 100 to 400 kPa.

**Table 1:**
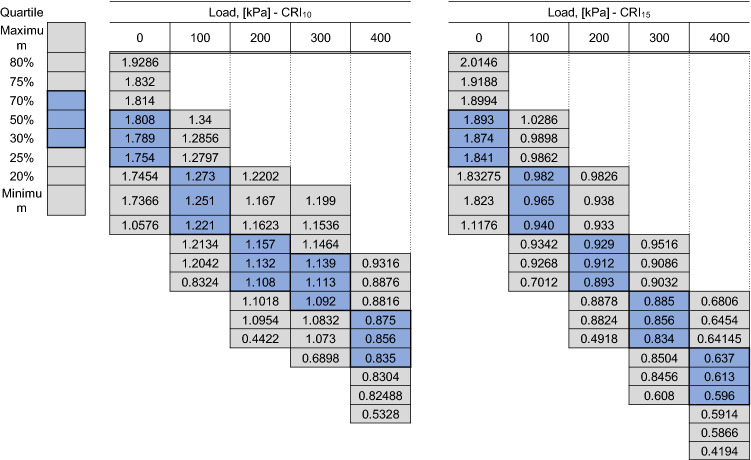
Distributions of cooling rate index.

The blue color corresponds with quantiles in the range of 20–80%.

## Conclusion

This study was performed to deduce residual stress in ground anchors using infrared thermography. Both passive and active methods were applied to determine the relationship between residual stress and anchor head temperature. The detailed conclusions are as follows.

- A passive method was applied to obtain the anchor head temperature in a large-scale experiment. The recorded IRT shows that the temperature increases and decreases with loading and unloading procedures.
- An artificial heat source was used to simulate active method, and the measured temperature was converted into a cooling rate index according to each load. The intervals of 10 and 15 min (CRI_10_ and CRI_15_) are shown to be reasonable time intervals for deducing residual stress.
- The number of pixels increases when load is applied in both passive and active methods because the strain is converted into thermal stress based on thermodynamic theory. Note that this study shows the IRT technique can provide residual stress to estimate the integrity of ground anchors.

## Data Availability

The datasets used and/or analysed during the current study available from the corresponding author on reasonable request.
